# Materializing architecture for social care: Brick walls and compromises in design for later life

**DOI:** 10.1111/1468-4446.12722

**Published:** 2019-12-19

**Authors:** Sarah Nettleton, Daryl Martin, Christina Buse, Lindsay Prior

**Affiliations:** ^1^ Department of Sociology University of York York United Kingdom; ^2^ Department of Sociology Queens University Belfast Belfast United Kingdom

**Keywords:** architecture, care homes, later life, materialities of care

## Abstract

This article reports on an ethnography of architectural projects for later life social care in the UK. Informed by recent debates in material studies and “materialities of care” we offer an analysis of a care home project that is sensitive to architectural materials that are not normally associated with care and well‐being. Although the care home design project we focus on in this article was never built, we found that design discussions relating to a curved brick wall and bricks more generally were significant to its architectural “making”. The curved wall and the bricks were used by the architects to encode quality and values of care into their design. This was explicit in the design narrative that was core to a successful tender submitted by a consortium comprising architects, developers, contractors, and a care provider to a local authority who commissioned the care home. However, as the project developed, initial consensus for the design features fractured. Using a materialized analysis, we document the tussles generated by the curved wall and the bricks and argue that mundane building materials can be important to, and yet marginalized within, the relations inherent to an “architectural care assemblage.” During the design process we saw how decisions about materials are contentious and they act as a catalyst of negotiations that compromise “materialities of care.”

## 1 INTRODUCTION

This article reports on an ethnography of architectural practice in the UK. The study examined the work of architects who design and develop buildings for social care, with a particular focus on the production of settings for dementia and later life. The aim of the research was to open the “black box” between the initial commissioning of buildings through to their delivery, in order to cast light on what happens during the design and construction stages of development. In addition, we sought to explore if, and if so how, ideas about care can be operationalized into design projects. In this article, we follow a rather prosaic design feature—a curved brick wall—while also examining the proposed brickwork of a building to reveal how, in one architectural project, attempts to encode values of care into a design were negotiated. In our analysis, we bring together literatures from sociological studies of material culture (Ingold, 2013; Shove, Watson, Hand, & Ingram, 2007) and conceptual innovations of “materialities of care” (Buse, Martin, & Nettleton, 2018; Fox, 2016). As a heuristic “materialities of care” makes visible inconspicuous aspects of material culture that are not routinely thought of as salient for care. It prompts exploration of the ways in which taken‐for‐granted materials infiltrate care practices and encourages analysis of the way material things are embedded into the socio‐political making of care settings. Through foregrounding the active role of materials in design work, and so “materializing architecture”, we offer a contribution that unpacks the micro politics of “care‐full” design (Boys, 2017) by revealing how designs for care are shaped not only by architectural form, but by the messier interplay between the inextricably interlinked processes of an *architectural care assemblage*.

Our data were generated during a UK based Economic and Social Research Council (ESRC) funded study called “Buildings in the Making: A Sociological Exploration of Architecture in the Context of Health and Social Care.” As the title of the study implies, our focus was on “making”: how people and artefacts collaborate in the production of buildings. The project was a response to Ingold’s call for more studies of “architecture in the making,” which he argues are rare (2013, p. 11). Although there are some sociological studies that examine the day‐to‐day work of architects (e.g., Imrie & Street, 2011; Sage & Dainty, 2012; Yaneva, 2009), to date there has been little research on the day‐to‐day practices of architects designing for health and social care. Drawing predominantly on data from one case study nested within our wider ethnography, in this article we aim to demonstrate how a conceptual shift in focus, from architectural buildings as “objects” to architectural buildings comprising “materials” that are continually in the “making”, helps us to think differently about architectural practices in the context of design projects for care. We present a case study that reveals how enthusiasm for “a” particular design feature fragments when negotiations inherent to its operationalization become contentious and “messy” (Turnbull, 1993).

We begin by introducing theoretical debates in studies of material culture informed by the writings of Shove et al. (2007) and Ingold (2013), before turning to describe our study methods. Drawing on our empirical data we then analytically reflect on the comings and goings of a “feature” curved brick wall, and the brick work more generally, of a care home design project. Our focus on the curved wall and bricks foregrounds knotty questions of negotiation, tension and compromise. The building we will focus on was never fully realized because of the results of an election which changed the political composition of the local authority that had commissioned the care home. Nonetheless, we will see how materials disrupt architectural design processes even where a building is not constructed.

## 2 MATERIALIZING MATERIAL STUDIES

Architects are invariably interested in the pragmatic, aesthetic, and symbolic significance of materials (Böhme, 2013). Contemporary debates have come to focus on “materiality,” rather than just materials, so as to explore the “immaterial” aspects of substance (Hill, 2006). Architectural scholars have explored materiality in terms of its sensory, atmospheric, political, phenomenological, and ecological dimensions (Löschke, 2016). These analyses complement both sociological histories (Cuff, 1992) and critical analyses of the architecture profession (Samuel, 2018). The focus on materiality resonates with recent sociological and anthropological studies that have begun to deconstruct relations between design and construction, creativity and controversy, and materialities and making (for a review see Verkaaik, 2016). To use the language of science and technology studies (STS) researchers, “materiality” foregrounds the agentic potential of materials (Jacobs & Merriman, 2011; Yaneva, 2009, 2012), and seeks to put “materials back into material culture” (Shove et al., 2007, p. 94).

Ingold, like Shove, argues that studies in material culture give an “overwhelming” focus to “objects” while neglecting the “materials” from which they are made (2013, p. 31). Of course, this distinction between objects and materials is fuzzy, but his analytic shift is important.

Ingold argues that “materials do not *exist*, in the manner of objects, as static entities,” but “as substances‐in‐becoming” (2013, p. 13). If we foreground “objects” of material culture (e.g. spoons, pans, or bowls) rather than what they are made of (e.g. wood, metal, or plastic), we underestimate their mutability. We also underestimate how “things” change in relational ways as they negotiate with other artefacts or elements, such as the weather, chemical reactions, or simply day‐to‐day use. In limiting our attention to objects, “the creativity of the productive processes that bring artefacts themselves into being … gets lost” and is therefore “swallowed up in objects made” (Ingold, 2013, p. 7). Ingold offers the concept of “correspondence” as an analytic for appreciating this relationality of architecture, where every material “becoming” arises through “a maze of trajectories” (2013, p. 31). We argue that turning the lens from objects to materials in this way provides a conceptual way in to explore the significance of architectural practice in the materialized assemblage of care (Buse, Martin, & Nettleton, 2018; Martin, Nettleton, Buse, Prior, & Twigg, 2015).

Shove and her colleagues point to the “potential of a more thoroughly materialised analysis of objects” (2007, p. 98). In their analysis of design practices, they aim to “describe material substances, like wood, aluminium and plastic, rather than discrete objects” (2007, p. 154), in order to produce a nuanced understanding of the translation of design projects in practice. In the context of architecture this means examining building as intrinsic to, rather than separate from, design where both are materialized activities. Ingold again:

Much can ride, in English, on the indefinite article. Building is an activity; it is what builders do. Add an article, however, and the activity is brought to a close. (Ingold, 2013, p. 47)

Informed by these insights, in the analysis of our empirical data we understood architectural projects as collaborative activities that comprise architects, designers, engineers, investors, builders, *and* materials weaving together more, and sometimes less, harmoniously. This is consistent with Shove et al.’s theorizing, in which design projects involve more than “deliberate human planning and decision making,” because they are invariably “materially implicated,” “exploratory,” “unpredictable,” and “surprising,” emerging “through and in the course of practical engagement between people and the material properties with and on which they work” (2007, pp. 61–67).

We extend these analyses through our attentiveness to “materialities of care” (Buse, Martin, & Nettleton, 2018) and, in what follows, we move on to examine what Latour (2004) calls “matters of concern” and Puig de la Bellacasa (2011) suggests are also “matters of care.” Latour proposes that sociologists should be alive to how things are assembled and stabilize into “matters of fact”. Puig de la Bellacasa argues that sociologists should not only look at how actors and actants sustain “facts” but should also explore their capacity to embed care. She writes that “to care signifies: an affective state, a material vital doing, and an ethico‐political dimension” (Puig de la Bellacasa, 2011, p. 90). And so, in our focus of building materials, we should be alert to how actors and actants are able to assemble and sustain “matters of care” as well as “matters of concern.” Empirical analysis has the potential to reveal how the heterogeneous elements of a design project for care come into being, by shadowing materials as their associated relations, networks, and tensions unfold (Latour, 2005). Architecture in this way is understood as an “assemblage,” both metaphorically and literally (Deleuze & Guattari, 1988). An architectural “care” assemblage (Fox, 2016) that is vigilant to matters of care is integral to the production and delivery of settings for later life. Exploring a design project in this way complements extant research on the architecture of long‐term social care and dementia (Buse, Nettleton, Martin, & Twigg, 2017; Nettleton, Buse, & Martin, 2018a; Nord, 2018; Nord & Högström, 2017; Del Nord, 2003; Torrington, 2006), and studies of health and social care architecture that examine how buildings are used, experienced or evaluated (Adams, Theodore, Goldenberg, McLaren, & McKeever, 2010; Gardner & Williams, 2015).

In sum, we are interested in following how materials are implicated in the architectural “making” (Ingold, 2013) of settings for care, tracing compromises made and observing how these alter the shape of buildings at different stages. Although the care home design project we concentrate on in this article was not actually built, materials are invariably implicated in the architectural processes. Early consensus between the various project stakeholders about the merits of the initial design features of the wall and textured brick work fractured and became fractious as the project progressed. In what follows we show in detail how materials are implicated in design and construction, and how different forms of professional knowledge (between architects, developers, construction professionals, care providers, clients, users, and others) become entangled through everyday materials. Bricks and brickwork are not conventionally considered significant for social care, yet here we show how such commonplace materials were deployed by architects to engineer care qualities into their design, so as to enact what Bille et al. refer to as “staging an atmosphere” for “collective affect” (2015, p. 3)*.* But first we offer some notes on our study methods.

## 3 STUDY METHODS

This article focuses on data from a single case study of a care home design that was nested in a wider ethnography of architecture for health and social care (2015–2018).^1^ The first stage of the study comprised 20 qualitative interviews with 26 architectural professionals (some interviews were with two participants) involved in design for care. Ethnographic research generated further data where the research team worked with nine architectural firms to observe their work in practice. The team then followed three design projects for later life care, each from different architectural firms, longitudinally for between 12 and 18 months. “Target ethnography” (Sage & Dainty, 2012) involved observing key moments including: design review meetings in architectural firms; wider project design team meetings (DTM) and construction site visits (variously attended by architects, care home operators, contractors, engineers, developers, and surveyors, amongst others); and public and user consultations. Working alongside the architects and their collaborators the aim was to generate a materialized thick description (Geertz, 1973) as we trailed the routes of materials through the intricacies of the design project.

The ethnography also involved analysis of documentary sources, such as minutes of meetings, planning documents, design and access statements, drawings, and design guidelines. An additional nine audio‐recorded discussions were conducted with architects involved with these projects as they talked though documentary sources and plans, and eight further interviews were undertaken with clients, developers, building contractors, and regulators. Interviews and case studies were numbered to ensure anonymity, and any names of individuals or companies used are pseudonyms. The research was approved by the University of York research ethics committee.

In this paper, we focus on case study three (CS3), a project we followed for 18 months. A specialist dementia care home had been commissioned by a local authority using a “design, build, finance operate” procurement route, and was financed, project‐managed and led by a developer (Ellis and Brown). This form of public private finance initiative (PFI) was the most complex model of procurement that we observed (Buse, Nettleton, & Martin, 2018; Nettleton et al., 2018a). The consortium led by Ellis and Brown were successful in their tender to the local authority, and included an architectural firm, a building contractor (ENT Constructions) and a third sector care provider. The architectural team was led by the director of the practice (Brian), who worked alongside a senior partner (Alan), and the project architect (Nick). All were registered architects.

Our data analysis comprised members of the research team reading interview transcripts, fieldnotes, and documents and then discussing them in detail. Interview transcripts and the field notes were uploaded together to the qualitative data software package NVivo, allowing the project researcher to comprehensively apply the coding scheme developed during our extensive discussions. Our analytic approach was consistent with “non‐representational ethnography,” defined by Vannini as “becoming entangled in relations and objects, rather than studying their structures and symbolic meanings” (2015, p. 320). And so, although we deployed conventional tools of data collection (interviews, observation and so on), we sought to cultivate a “material imagination” (Richardson‐Ngwenya, 2014) when reflecting on how bricks and a brick wall had the capacity to effect change and rework the architectural making.

## 4 BRICK WALLS AND MATTERS OF CARE

Case study 3 (CS3) was a care home project designed by an architectural firm which, as part of a wider consortium, had won a competitive bid to develop the property for a local authority. The architectural practice has an established portfolio of work in the social care sector and most especially for later life care and for people living with dementia. The design for this care home had been developed in response to a brief that emphasized the importance of embedding dementia‐friendly design principles (Dementia Services Development Centre, 2011), while ensuring that it would also be financially viable. The concept design submitted by the consortium presented a two‐storey care home with 60 bedrooms, split into smaller wings or “households” to create more domestic scale dwellings. The design featured a community “hub” with a shop, pub, cafe, and hairdressing salon. There was an emphasis throughout the bid documents on materials and design features that would ensure a “domestic” feel.

One architectural feature of the proposed care home was a curved brick wall with distinctive brickwork at the entrance of the building. In the bid, the feature wall was described by the architects as creating an entrance that would be “welcoming” and “homely” and “encourage a sense of belonging.” The materials in the documentation are depicted as “brick of two colours” (red and white), in order to create a “residential” façade while “being a high‐quality modern design.” The value of the feature was reiterated throughout the *Design and Access Statement*:

The curved wall by the entrance will present a pattern using the two colour bricks, inviting visitors and staff towards the entrance. … The laundry wall facing towards the access to the site has been designed in a way that will mark the access of the building inviting people towards the entrance. The radius of the curve is wide enough to avoid “faceting” of the bricks and creating a smooth elegant curve. The pattern will be created using the two types of bricks used throughout the building adding to the dynamic nature of the curve and enhancing the impact of the wall.

The brick wall was presented as an architecturally significant feature, not just aesthetically—“a smooth elegant curve,”—but also meaningfully, “inviting people” towards the home.

After the award of the tender a series of project DTM were held to develop the details of the design, including the services, structural issues, planning matters, sustainability requirements, and materials. During a DTM where the developers, building contractors, and care home providers discussed the design, Brian said that he “doesn’t want the main focal feature of the building to be a utilitarian wall.” Pointing to the plans laid out on the table he talked about the value of “exploring the materiality of the wall” and how the brick patterning would “connect to the patterns in the paving” in ways that would “draw people into the entrance” (Fieldnotes, summer 2016). The thinking behind the curvature of the wall, combined with two types of bricks, was to introduce movement—“the dynamic nature of the curve”—so as to off‐set what might otherwise communicate an austere, “utilitarian” façade. Brian’s comments echoed Kraftl and Adey’s (2008) findings where they described how architects used design features “to kindle certain capacities for inhabitation” in order to “intentionally shape the experience of, and emotional response to, a place through the material environment” (2008, p. 225). Our data show how what to those outwith architecture might consider to be mundane building materials were mobilized to invoke a *domestic* feel, so as to avoid the care home looking like an institution. The architects saw the brick as having the potential to foster an appropriate atmosphere and affect (Bille, Bjerregaard, & Sorensen, 2015), indicative of a phenomenological architecture (Paterson, 2017). This finding touches on a recurrent theme throughout our wider study where architects told us how important it is that care settings are “domestic” and “homely” in scale so as to avoid any suggestion of the institutionalized care that they associated with a bygone age (Buse et al., 2017; Nettleton et al., 2018a, 2018b).

Shelley, a representative of Ellis and Brown (developers), told us during an interview that the curved brick wall was a design feature that had been appreciated by the client and that this type of architectural detail communicated a sensitivity to care, all of which had contributed to the success of the bid. She explained the importance of working with architects who are able to embed care into the design narrative through, for example, “the use of colour,” and “having things you can touch that are tactile.” Although at times she drew on negative tropes of ageing, such as references to residents “wandering” that would jar with contemporary social commentators (Higgs & Gilleard, 2016), she nevertheless articulated a design narrative that was attentive to materialities of care. For example, during one project DTM she challenged the contractor’s assumption that “modern” design and interiors would not be suitable for older people when she said: “you can’t assume they want chintz, why should they have chintz and thick curtains” (Fieldnotes, DTM, spring 2016).

We begin to see how materialities of care give rise to tensions. Although there had been agreement about the merits of the wall and the brick work during the tender process, when it came to the construction, the initial enthusiasm of ENT Contractors waned. As Shelley from Ellis and Brown explained:

[the builders] are always going to come from the perspective “the architect’s drawn something a bit stupid” that they don’t think is easy to build. They just want a rectangular box often and we have to say, “that’s not what’s going to win us schemes”. (Interview developer representative CS3)

Curved brick walls are awkward. As Unwin writes in his seminal textbook, “it is not easy to construct curved form from rectangular bricks,” but nonetheless he argues such walls are attractive because circular forms can “accommodate the social geometry” and so encourage human interaction (2014, p. 161).

A curved wall featured in another of our case studies (CS7) where we followed a building for extra care (that is housing that allows residents to have independence while having varying levels of supported care in place if needed). In this dwelling, the curved wall had been constructed despite, as the project architect put it, the challenges of the “buildability of curves which for the contractor was a bit of a nightmare.” This architect was also pleased that the extra care facility was built from “real stone from the local area,” and so spoke to the vernacular suggesting a rootedness to place. She therefore articulated a materialized narrative of care.

The windows that in these dormers, they are all clad in real zinc, and the council wanted this triple glazing in VELFAC, which is aluminium so it’s not UPVC, and it’s got this nice wood finish internally. So all these little things mean that it takes it away from what people associate with affordable, which is really good … Older people who don’t have a lot of money shouldn’t have to put up with rubbish. (On site walking interview architect CS7)

For this architect ideologies of egalitarianism were materialized where bricks, stone, aluminium, and wood serve as what Puig de la Bellacasa refers to as “matters of care” (2011). The haptic quality of the building was understood to be integral for its affective capacities and for enabling a respectful environment for future inhabitants.

For architects in CS3 the quality, type, and layout of the bricks were important. At the outset, they envisaged that they would use clay bricks but, as the project progressed, they compromised with the contractor’s preference of cement bricks. Project architect Nick told us towards the end of our fieldwork that they had been “very nervous about the cement bricks because they tend to discolour” but he described how the contractor convinced them otherwise:

Apparently they’ve found a way to protect the colouring of the bricks from UV radiation. So yeah, we’re getting there, and they also took us to see a care home in [named town] that had these bricks, and they actually look alright, so I’m kind of happy with that. (Interview project architect).

But our data show that this was no easy compromise. Early on in the project we observed how Nick had been keen to get the bricks “signed off” by the wider team. Prior to a project DTM he submitted costings for his preferred bricks to Jeff the building manager (ENT Constructions) and then followed this up in the meeting:

*Nick*: Did you receive my update on the bricks?

*Jeff*: Yes, white bricks. It was £750 per square metre … which is over twice what we normally look at.

Normal brick is £250/£300 for an external wall.

*Nick*: (*starts doing some calculations …*): How many bricks per square metre?

*Jeff*: It is an expensive brick.

(Fieldnotes design team meeting (DTM) spring 2016)

Jeff closed off the discussion and when he saw Nick scribbling cost calculations he commented that no amount of mathematical gymnastics would make the proposed bricks affordable. We see in these data how we are already beginning to move from an objectified image of the brick façade to the correspondence between the human and non‐human participants (Ingold, 2013), offering an example of the relational dynamics of architecture where “things” change in their making. Nick’s efforts to use materials as a means to encode a quality of care into the design had prompted concerns. In this humdrum example, the bricks become what Latour calls “matters of concern” (2004); “things” acting as “issues” and provoking tensions. As we go on to see, Nick would have to rethink the bricks, which have become “social” (Yaneva, 2009).

## 5 BRICK WORK AND REMAKING ARCHITECTURE

During CS3 we observed design review meetings (DRMs) held in the offices of the architecture practice. We saw how Brian (director of the firm) and Alan (senior associate architect) appraised the progress of the project. Nick (project architect) and Jess (an architectural trainee) displayed printouts of computer aided design (CAD) drawings, plans, and elevations of the care home. The format of the DRM was reminiscent of the tradition of the “crit” that we had observed during our wider project, when we sat in on student assessments in architectural schools (Webster, 2004). There, students presented their plans and elevations to peers, staff, and external practitioners and the feedback process was reminiscent of professional rituals that, as Lipstadt (2003) points out, inculcate an architectural habitus. During the CS3 review, the team discussed a range of options for the layout and shaping of the wall. Nick proposed options for addressing the “problem” that had resulted from the contractor’s rejection of his proposed bricks.

Nick said “how to move forward with the building depends on the quality of bricks they are using. One idea is maybe popping out the parapet—three bricks—which could bring a different element to it”. Nick gestured to an image of the exterior and the windows which he said, “are looking a bit plain” and “the question is can we do anything else with the brick without making it a lot more expensive? Maybe sinking a brick 25ml or taking a brick out 25ml that could influence the brick bringing additional texture to the way the windows work.” He said he “wants to add more texture—something, along those lines” as he gestured to the drawings “without having to go to special brick.” He took one of the drawings down from the wall and explained to Brian “the bricks are being taken out 25ml, so here the bricks are poking out 25 ml, and this side the bricks are poking in 25 ml.” Brian responded this “is going too far” and they should “keep it simple.” Alan suggested that they “leave that sort of detail for the curved front wall.” At this Nick questioned “really?” and Alan said “yes it is already enough.” (Fieldnotes, DRM, spring 2016)

Again, we see here architecture as correspondence (Ingold, 2013); although the architects do not directly work with bricks they do consider how they “will work.” This term “will work” was ubiquitous throughout our data, implying the continual effort, activity, and efficacy of materials. Perhaps they were thinking about the famous architect Louis Kahn, who is reputed to have said that even bricks will want to “be something,” urging his profession to engage in dialogue with them (Lesser, 2017). Materials are not presumed to be inert but relentlessly “working” in correspondence with other materials. But, as we have seen, Brian and Alan were not fully convinced by Nick’s plans when they suggested that he was “overcomplicating” the building. Perhaps sensitive to the contractor’s preference for the workability of a “square” box (as noted in the developer’s quotation cited above), Brian commented that “presumably ENT Constructions will have a view about that,” implying they will query the cost implications of the proposed non‐conventional brick layout.

Later during the DRM Nick asked “Is it really more complex?”, to which Brian replied “of course it is.” The tenor of this discussion reflects the findings of Sage and Dainty’s ethnography of architects where they observed a “curiously submissive power dynamic” infusing professional practice (2012, p. 9). Other artifacts associated with design were also brought to the fore in our data: we saw how play of the materials of construction worked alongside other materials in the architectural office, such as the CAD drawings, pencils, and sketches that have been written about elsewhere (Ewenstein & Whyte, 2007; Henderson, 1999). All actants in the DRM served as intermediaries as the designers reflexively co‐constructed proposals. In architectural practice, we saw too how hand drawing as a way of thinking and communicating continued to overlap with newer practices of CAD (Groleau, Demers, Lalancette, & Barros, 2012). Indeed, throughout the meeting Brian annotated and traced over Nick’s printed plans, enacting the hierarchical relations of the team.

Brian started redrawing the area on the tracing paper whilst saying, “if you did that the reception can have a window”, and “we’d just get back the simple geometry”. He said “what I’d like is that the geometry be absolutely rectangular everywhere except for that curved area, so that the curved line has its own big specialness. And it’s made more special by it being the only thing that changes, you know what I mean?” Nick agreed and commented: “so you get the kind of calm rhythm of the orthogonal geometry, just life going through the building, and then you get this one snaky beautiful thing”. Brian said: “I think it will look better”. To which Nick replied “okay”, and Brian said, “so that’s the curved thing which is lovely, and it’s still lovely, it’s survived.” (Fieldnotes ,DRM, spring 2016)

This again echoes Sage and Dainty’s findings where senior architects display “an embodied, or tacit, knowledge, based on decades of enjoyable success of finding a balance between what architecture can do aesthetically, culturally, emotionally, commercially and functionally” (2012, p. 9).

The exchange was hierarchical yet participatory, indicative of what Murphy calls “architectural imagination” where creativity is situated and communal, and designers “must constantly imagine what they are all talking about” (2004, p. 270). Imagination, he argues, is a “joint activity” facilitated through sharing drawings, plans, and sketches during “continuous meetings.” What “emerges in the moment of interaction,” Murphy argues, “is a temporarily imagined building” (p. 270), but also a temporally imagined building through each iteration. Each version of the design is a response to challenges or possibilities and will undergo a myriad of further changes in the face of financial constraints, local residents, environmental considerations, building regulations (Latour & Yaneva, 2008) and, for these architects, a sensitivity to the anticipated users of the building.

During the DRM the senior architects discussed other care homes and health care projects where brick walls “have worked.” Alan recalled an “even more subtle” example of a health centre:

“All we did there was just changed the mortar, changed the joint, from a slightly recessed to a flush and it just makes a huge difference, and it is almost similar to what you are showing here” [as he pointed to Nick’s drawing]. Brian suggested Alan digs out an image of it because “Nick needs convincing.” Alan said he liked this idea because “we’re playing within their rules then aren’t we? Because you know they are saying you can only have one brick and you can’t do fancy stuff, well all they need to do is, when they lay the bricks, is rake the joint out or trowel it out flush, and it’s literally the guy who is laying the bricks that is making the difference, it’s nothing else.” (Fieldnotes, DRM, spring 2016)

This exchange was mildly teasing so as to “convince” but not undermine Nick. Moreover, through the reference to the builders’ resistance to their “fancy stuff,” Alan also played on their collective professional architectural habitus, and their educational and cultural capital (Stevens, 1998), implying that as architects they can appreciate the socio‐historical significance of bricks (Campbell, 2003). This relates to other examples of the use of brick in later‐life care settings, such as, for example, the iconic brick‐built *Guild House* in Pennsylvania, designed by Robert Venturi and Denise Scott Brown in the early 1960s to accommodate older people on low incomes (Upton, 1998, pp. 244–245).

We see this as a nuanced exchange especially when we consider the architectural precedent of using mortar joints to add texture, quality, and atmosphere in the brick buildings of the Swedish architect Sigurd Lewerentz (Flora, Giardiello, & Postiglione 2013), whose reputation within the profession is highly prized because of the refined use of simple materials to create calm and atmospheric spaces (St John Wilson, 1997). Within standard UK practice, the mortar joint is expected to maintain a consistent standard application by builders, with any deviations reconciled through cutting or adjusting the brick. In Lewerentz’s church at Klippan in Sweden, this relation was reversed, with the architect specifying no changes to the brickwork, but all deviation to be resolved through variations to the mortar (McVicar, 2010). Reversing the traditional hierarchy in UK construction between brickwork and mortar work, Lewerentz’s church had the additional effects of closer working on site between architect and builder, an increased recognition of the skill brought by construction workers (an issue we return to below) and an invitation to visitors to “look at bricks as if they were a new material” (Caruso, 2008, p. 79).

In our example, the option of varying the mortar joint was believed to offer an effective and low‐cost solution to the use of expensive bricks. Alan’s suggestion that a solution to the expensive brick could be found in the laying of the bricks and mortar relies on the “work” of bricklayers, which prompts further contingencies. This was illustrated in an account from another architect during an interview in our wider study. He recalled an example where the brick of a care home project had not been laid correctly.

“You take bricks from different pallets so it’s all mixed up. But the bricklayers didn’t do that here, and no one noticed until the scaffolding came down and it’s pretty obvious that there’s different bands of brick, all slightly different shades. Same brick but because it comes out of the brick kiln at different times it all is slightly different. Look at the building over there, all those bricks are slightly different, but there is darker bricks and lighter bricks all mixed up, so you don’t really notice. But you can imagine all these lighter bricks up to a certain height, then all the darker bricks were another height, because they just took them from one pallet at a time. … Two years later it’s weathered and it’s not as noticeable now, but when that scaffolding came down it was really noticeable.” (Scoping interview 8, architect).

We know that brick can be malleable; in this example, we were told how the weather had gradually smoothed over the troubled wall and bricks became inconspicuous. In another of our case studies, the subcontractors “messed up” and the brickwork judged by the site manager to be substandard had to be redone causing delays and additional costs (CS9).

Thus bricks “work” in ways that can be capricious, especially in relation to things such as weather, heat, chemical reactions, mites, plants, and builders (Hill, 2013; Mostafavi & Leatherbarrow, 1993). Working and making are in effect the same (Ingold, 2013); both unpredictable, exploratory and often surprising (Shove et al., 2007). Bricks carry on “working” after they have been laid, as their varying properties (cement, clay or concrete) are mutable and can invoke sensory affect. Indeed, we saw Alan and Nick become quite quixotic when Brian suggested that they do further research to scope types of brick for the project.

Alan said that “down in [Named] County they have done it Flemish bond” and added that “because of the way [the bricks] are cooked in the kiln all the long sides are red, and the ends are blue and so you turn the brick round” and “the ends are a different colour but it is the same brick”. Nick remarked “that’s cool.” (Fieldnotes, DRM, spring 2016)

Here we see an attachment to materials that have scope to enact care, through a heightened understanding of the salience of materialities for nurturing atmospheres and for adding texture to spaces for affect (Bille et al., 2015). In the DRM, Brian listened to his colleagues’ reflections on kilns, the craft of brick making, and the merits of Flemish bond, but then brought them back into line, when he pointed out that they “don’t need to have special brick if we follow Alan’s suggestion of the pointing, where you are simply setting them in and out by most probably 10 mls.” In Brian, we can sense a juggling between the architect’s empathetic appreciation of craft and the affective properties of the materials that can encode care through the introduction of texture, set against what is financially acceptable. The team acquiesced but were still cautious about the choice of bricks. Alan pointed out that if they use “red bricks they will have to be clay, I have a thing about concrete red bricks because they fade, they go pink, it’s the pigments they use, and we don’t want to have a salmon pink building in twenty‐five years’ time!” But as we saw above, the building contractor managed to persuade them that new material technologies would ensure that concrete bricks would not discolor and so the architects had agreed to forgo their preference for clay.

## 6 MATERIALS AND MESSY PRACTICES

While the DRM meetings took place in the architectural office, DTM took place adjacent to the case study site. Builders, developers, electrical engineers, mechanical engineers, care operators, and fire safety officers were amongst those who attended these meetings. Here Nick outlined the plans for the patterning and/or pointing which he proposed as a “no cost solution.” ENT Constructions were not convinced.

*Adam* (*ENT Constructions*): Who did you talk to? It will cost.

*Shelley* (*Ellis and Brown Developer*): It adds extra complexity.

*Adam*: Is it a random pattern?

*Nick*: It is a line of bricks pulled out 10 ml.

*Jeff* (*ENT Constructions*): It will cost more.

*Nick*: Mentions “plan B” the “pointing of mortar.”

*Jeff*: “Anything that steps away from the ordinary will add cost.”

(Fieldnotes, DTM, spring 2016)

“Away from the ordinary” implied a bespoke crafting and attention to detail that rubs up against costs. Adam, a member of the construction team, explained why during a subsequent interview:

“If you’ve got a red brick building which is just straight up red brick, no discrepancy or flair in it, and you can stick a graduate on there with a couple of not too experienced bricklayers and they will knock it out no problem. As soon as you start going ‘well, actually I want white bricks here and here I’m going to set it out three bricks in from the edge and then it will repeat every four or so’, suddenly people have to think about it a little bit more. Although the architects are thinking, well ‘that’s surely not an extra cost’, unfortunately it is. It increases the time amount, because instead of just laying every brick they’re having to go and get another white brick and then they’re thinking about where it goes and if they put it in the wrong place the architect is going to say that brick needs to move.”

(Interview building contractor CS3)

The additional cost was therefore not only about the materials themselves, but also about the practicalities and contingencies of bricklaying, something the contractor felt was not appreciated by the architect. We see here an exemplar of wider tensions between construction and design, builders and architects, design and buildability (Imrie & Street, 2011).

During this same DTM, and as a result of an intervention from Shelley the developer when she said that they would be “mad not to do something,” it was decided that the curved brick wall with patterning would be retained. And so the feature wall survived although, as Nick later told us, the building contractor subsequently reduced the brickwork across other facades as part of a wider set of value engineering (VE) reviews.

Another VE exercise was us having to flatten a lot of the façades to reduce costs. The transitions between one colour of brick and the other ended up happening on the same plane, and we didn’t like that, because it just tends to look cheap. We wanted to do it all around the building, but we weren’t allowed to do so, so this was just left on the front, the entrance façades. But the rest, it’s going to be flat. Which is a bit unfortunate. (Interview project architect CS3)

Through the reference to brickwork looking “cheap,” Nick implied that this undermined the potential to engender a positive sense of value and quality for the anticipated residents of the home. Nick’s enthusiasm for particular bricks and for the curved brick wall may also have been tied to professional pride and esteem, as retaining the initial design concept was a marker of his creative skill and architectural inventiveness. Brown, Kornberger, Clegg, and Carter (2010) suggest this is a key feature of the negotiation on identities within architecture firms. However, it seemed to be about more than this. The architects’ dedication to the brickwork throughout the project exemplified what Caruso calls acting in architectural “good faith,” whereby architects can act critically in the construction process through insisting on upholding qualities of materiality, design, and vernacular sensitivities, in spite of market pressures (2008, pp. 49–53). We have seen from the outset how these architects felt it important to avoid a “utilitarian” feel and instead stage a materialized atmosphere of care (Bille et al., 2015). The curved wall was an architectural manoeuvre to “inject life,” “movement,” and “calm rhythm,” in order to invoke dwelling *with*, rather than merely existing *in*, a place.

We saw evidence of this commitment to care elsewhere on the project when Nick had to compromise on other materials. For instance, Nick’s choice of wood window frames for a “warmer homely feel” was replaced with UPVC composite frames, in order to meet the client requirements for a high score on a measure of sustainability as set out in the BREEAM assessment criteria (see https://www.breeam.com/) (Fieldnotes, DTM, spring 2016). In fact, the windows of the residents’ rooms were part of a wider tussle. The bay design with a deep sill was intentional because, as Nick explained, it would have created space for residents to “have their own stuff … bits and pieces that connect to your previous life and to your family.” As McCarthy found in her research, bay windows are an integral element of cultural ideas of the “ideal home” for many in the UK (2018, p. 972). But the bay design was resisted by ENT Constructions who preferred a single rather than three‐paned window because it would be quicker and cheaper to construct. Neighbors, too, during a planning consultation, opposed the bay designs, arguing that these would comprise their privacy. But the architects stood their ground, imagining the space to be crucial to the lived experiences of the residents and giving scope for ongoing processes of homemaking.

Our focus on materials and making therefore reveals how the competing priorities of architects and building contractors are entangled throughout the processes of design and construction, supporting Ingold’s thesis that the creative work of the building is not purely “concentrated in the process of the design” (2013, p. 47). Imrie and Street (2011) also argue that the separation of architect and builder has been overstated. In his analysis of the construction of Gothic cathedrals, Turnbull (1993) challenges an assumption that these spectacular buildings “must” have been designed, and then designs passed on to obedient builders. Instead, then and now, buildings are better understood as “experimental laboratories” (p. 322), where masons and architects work with plans through a series of “local and ‘messy’ practices” (Turnbull, 1993, p. 317). Architectural projects for care similarly are not designed then made, but are assembled through the practices of contractors, builders, engineers, care providers, and materials. Within this “architectural care assemblage,” where we see a series of challenges and compromises or “messy practices” there is a danger that the values and vision of care inscribed in the initial design plans and statements may be diluted. As we noted in our discussion of Lewerentz’s church (McVicar, 2010), it is important that all members of the design and construction team are cognisant of and committed to creating a space for “care.”

In another case study (CS6), the contractor joked to us that “we build it then Arthur [the architect] draws it.” Of course, designs were in place at early project stages to secure planning approval, prepare costs, contracts, and so on, but there is substance to the joke. Contractors modify instructions in situ. On one occasion, the builders built a wall with bricks that were conveniently to hand, rather than those detailed in the design plans. On seeing this, during a site visit, the contractor and architect agreed that the wall would still “work” and together they would mollify the client. Designs rarely go according to plan, as the site manager on this case study put it:

“things look fine on a flat piece of paper, but out on the coalface, in the real world, it doesn’t always work, so we’ve got to make it work on the hoof!” (Interview site manager CS6)

The freedom for the contractor to modify the design in situ reflects the fact that in CS6 there were long‐standing relationships with high levels of trust between the architect, contractors, and client, who had worked together on many care homes and crucially shared a vision of, and a commitment to, the qualities of care. This was in contrast to CS3, where contractual relations were complex and formal. It also points to the contribution that builders can make to crafting the atmospheres of places. Materializing architecture for care needs the commitment not only to the proposed architectural form, but also to the “detailed crafting of materials and spaces” (Boys, 2017, p. 157).

## 7 CONCLUSION

In this paper, we have sought to “materialize architecture” by following the trajectory of a curved brick wall and brick work in a care home design, and we have cast light on the social processes of architectural work as they unfold between the commissioning and construction of a building. We have argued that prosaic materials as well as design details are significant to creating spaces for care. In the case study presented in this article, we saw how at the initial tender stage there was a consensus between the developers, contractors, clients, and designers about the merits of the curved brick wall, and that colored and textured brickwork would engender a welcoming, hospitable feel. However, once the project team came to focus on the making, the initial unanimity began to unravel. The “*architectural care assemblage*” destabilized and sustaining it involved tenacity and creativity. The brick wall and proposed brick work acted as catalysts and created tensions. We saw how a commitment to a “materialities of care” (Buse, Martin, & Nettleton, 2018) comprising the immaterial qualities of things such as texture, aesthetic, color, atmosphere, and feel fractured, as these qualities became “matters of concern” (Latour, 2004), and also risk becoming marginalized as “matters of care” (Puig de la Bellacasa, 2011). This materialized analysis of design work therefore demonstrates how the agency of mundane materials complicates the construction of spaces for care. The curved wall and the brick work was chosen to encode values of hospitability and affective care, yet they were resisted in the context of anticipated resource constraints.

Attention to materialities illuminates the level of mundane detail at which compromises around care are assembled during design and construction. Territorial disputes between architects and builders about the merits and demerits of particular bricks may evoke underlying ideas about architectural habitus and professional integrity (Stevens, 1998). However, taking a wider view, they also point to the situated contingencies and priorities that all contributors bring with them to the project. The fact that CS3 was never built reflects some of the problems with PFI models of funding in health and social care sectors (Jones, 2018; Pollock, 2005). Our example shows how buildings designed to be sensitive to practices of care in later life may be undermined as design concepts become design concessions within funding envelopes and the use of particular procurement models.

By limiting our focus to the brickwork in this case study, we have understood the architectural feature of the curved brick wall not by imagining it in a completed, definitive state but, rather, by tracing its materiality through the earlier and ongoing struggles between architects and others and, indeed, between fellow architects. In doing so, we have come to a better understanding of the interlocking technologies, regulations, and relations that underscore architecture as a collaborative practice (Kraftl & Adey, 2008). An attention to architecture as a materialized activity, rather than deferring analytically to the designed or eventual articulation of its buildings, opens up the potential of understanding architecture, and its making, as “a process of correspondence” (Ingold, 2013, p. 7) and, in this case, its contribution to building new spaces of care. Brick work enabled us to recognize the flux by which our built environment comes into being (Shove et al., 2007) and, moreover, hold onto a sense of its becoming (Gieryn, 2002). Doing so prompts an understanding that, as well as an art of creating space, architecture is a profoundly temporal practice, with the meaning of its artefacts never stable, but always mutable and dynamic. Through materializing architecture, we contribute to an understanding that leads us to know architecture as involving more than just architects and designers, but a host of other human and nonhuman contributors—and to recognize that all have a pivotal role in an “*architectural care assemblage”* over the fullness of time.

## NOTE

1 For further details of the study see the Buildings in the Making project website: ://buildingsinthemaking.org.uk/about/
